# The experience of people with multiple sclerosis who receive occupational performance coaching

**DOI:** 10.1016/j.pecinn.2025.100418

**Published:** 2025-07-03

**Authors:** Niloufar Malakouti, Dorothy Kessler, Marcia Finlayson, Samantha Stephens

**Affiliations:** a School of Rehabilitation Therapy, Queen's University, Louise D Acton Building, 31 George Street, Kingston, Ontario K7L 3N6, Canada; b Department of Neurology, Medical University of South Carolina, Murray Center for Research on Parkinson's Disease and related Disorders, 208 Rutledge B, Charleston, SC, United States; c The Hospital for Sick Children, Toronto, Ontario, Canada

**Keywords:** Rehabilitation, Self-management, Occupational performance coaching, Multiple sclerosis

## Abstract

**Objective:**

To explore how Occupational Performance Coaching (OPC) influences self-management in the daily lives of people with Multiple Sclerosis (PwMS).

**Methods:**

A qualitative study involving 10 PwMS who underwent 6 sessions of telephone OPC over ten weeks. Interpretive description was used as the methodological approach. Participants were interviewed pre- and post-intervention, with thematic analysis performed on transcripts.

**Results:**

Pre-intervention themes included resisting MS, living with MS, ongoing challenges, and strategies. Post-intervention, the theme of resisting MS dissipated, with emergent sub-themes of planning ahead, being consistent, and talking about the plan. Participants reported reduced resistance towards their condition, a shift in their focus from problems towards solutions, and an enhancement of existing strategies and/or development of new strategies used to overcome ongoing challenges in living with MS.

**Conclusion:**

OPC may facilitate a shift in focus towards solutions and enhance self-management strategies in PwMS.

**Innovation:**

This study highlights OPC as a promising and innovative approach for addressing the self-management needs of individuals with MS, emphasizing its potential to enhance meaningful participation by fostering effective coping strategies and proactive attitudes.

## Highlights

• Occupational Performance Coaching promotes solution-focused strategies for MS management.
• Occupational Performance Coaching helps individuals with MS adopt realistic and actionable strategies.
• Participants gained self-management skills, including learning to plan ahead.
• Occupational Performance Coaching helps shift focus from MS-related challenges to practical solutions.

## 1 Introduction

Multiple sclerosis (MS) is an inflammatory neurodegenerative disease. Worldwide, an estimated 2.8 million people were living with MS in 2020, reflecting an increase of more than 20 % since 2013 [1]. People with MS experience a wide range of symptoms including, but not limited to, fatigue and loss of motor function [2]. Symptoms of MS differ significantly from person to person, can change over time, and can significantly limit the participation of individuals in their daily activities and functions. Living with MS can, therefore, be both physically and emotionally challenging [3]. Accordingly, PwMS have described their participation in meaningful activities as a constant struggle especially as the disease progresses [4]. The substantial impact of MS on the health and well-being of those affected underscores the importance of comprehensive support to enhance their quality of life.

Adjusting to the diagnosis of MS is a life-changing experience, requiring individuals to come to terms with the long-term consequences of the disease and make necessary adjustments to their personal and social lives [3]. Self-management becomes crucial for PwMS to better adapt to the illness and manage their day-to-day lives, as well as reduce the risk of secondary conditions such as depression, anxiety, and sleep problems [5]. Self-management involves acquiring and practicing skills related to medical, role, and emotional management, enabling individuals to effectively cope with the challenges of their condition [6]. Interventions that promote self-management have shown positive effects on PwMS, including improved medication adherence, fatigue management, and overall well-being [5,7].

In addition to self-management interventions, research has explored psychotherapeutic approaches that promote values-driven behavior despite ongoing challenges, with Acceptance and Commitment Therapy (ACT) being extensively studied. Meta-analyses in chronic pain populations show ACT effectively enhances psychological flexibility and engagement in valued activities [8]. Preliminary studies in people with multiple sclerosis (PwMS) also report benefits for improving quality of life, resilience and well-being [[9], [10], [11]]. However, none of these studies have directly focused on participation goal-setting across varied daily activities or on coaching strategies to build self-management skills. As a result, there is still a lack of interventions specifically aimed at guiding PwMS in setting and achieving meaningful participation goals.

Occupational Performance Coaching (OPC) is an individualized strength-based, solution-focused and goal-oriented intervention that holds promise for supporting self-management in PwMS [12]. OPC originated from the work of occupational therapists with parents of children facing participation challenges [13]. It involves a collaborative partnership between the practitioner and the client to support achievement of client selected goals related to participation in valued activities. The practitioner provides emotional support to create a high-trust relationship and facilitates knowledge sharing as the client is guided through reflective questions to identify and work towards meaningful goals [14]. OPC emphasizes autonomy, with the active involvement of the client in exploration of solutions, decision making and taking actions towards achieving goals [14]. Through the process, clients develop knowledge and skills for self-management that can be applied to future goals.

While there is growing evidence of the potential benefits of OPC for self-management in people with chronic conditions [[14], [15], [16]], the specific impact of OPC on self-management among PwMS remains unclear. The dynamic nature of MS, with symptoms fluctuating over time, requires distinct self-management skills and strategies to adjust goals based on different situations and apply knowledge to different daily activities [17,18]. Therefore, this study explored the experiences and perspectives of individuals with MS in managing their condition before and after receiving OPC to examine how OPC influenced their self-management skills and ability to set and achieve participation goals. By using a qualitative research design, this study sought to provide an understanding of self-management practices and challenges of individuals with MS and explore the potential of OPC to improve self-management practices.

## 2 Design and methods

We used an interpretive description approach to address the above research objectives. Interpretive description draws from a range of analytic approaches to create ways for understanding individuals' experiences. In order to generate usable knowledge, researchers need to understand each case in detail and identify the common themes and patterns that might exist among these individual cases [19]. Accordingly, this approach was chosen for its ability to provide rich, contextual insights into the complex, subjective experience of the participants undergoing the intervention. Furthermore, this approach enables the capture of nuanced changes in participants' perspectives and experiences pre- and post-intervention, facilitating a deep understanding of the intervention's impact.

### 2.1 Sample and recruitment

This study recruited 10 participants who were in the intervention arm of a randomized controlled trial (RCT) of OPC with PwMS [20]. The main RCT study included individuals with MS who were over the age of 18 and were able to participate in coaching in English. Participants were excluded from the study if they had serious cognitive impairments based on Short Blessed Test [21] or severe depression measured by PHQ-2 [22] which would limit their ability to engage in the intervention. Participants were also excluded if they were receiving life, health, or executive coaching by a certified coach.

Qualitative and interpretive description studies in MS commonly employ small purposive samples to achieve thematic saturation [[23], [24], [25]]. Methodological guidance further suggests that interpretive description studies achieve adequate depth with 10–12 participants [25]. The sample of 10 participants in this study therefore aligns with established qualitative precedents and ensured sufficient depth to meet the study objectives.

To minimize potential contamination from concurrent behavioral interventions, we screened all candidates for active psychotherapy. Individuals who were currently engaged in any form of formal psychotherapy or behavior-focused intervention (e.g., ACT, CBT, or other coaching/therapy) were excluded from participation.

### 2.2 Data collection

Semi-structured interviews were conducted over the telephone before the start and following the completion of the coaching process to explore the participants' perceptions of their self-management skills before and after receiving OPC. Interviews were conducted by the first author, who also provided the OPC intervention. Prior to the intervention, questions were asked to gather information on participants' experiences of living with MS and how they managed challenges related to MS. A post-intervention interview was conducted using the same questions to explore how OPC may have influenced their abilities to manage living with MS. More specifically, participants were asked to comment on how they approached challenges of living with MS, including the processes of making decisions, solving problems, setting goals and finding and utilizing resources. Follow up questions were asked to explore the factors perceived by participants to facilitate or hinder their self-management. To minimize potential bias resulting from the interventionist conducting the interviews, care was taken to focus the questions on how participants were managing and not on their perception of how the intervention did or did not help. Because most participants started to talk about their goal achievement and changes they had made as a result of OPC in their final coaching session, transcripts from this session were included with the post OPC interview data. Interviews ranged from 20 to 50 min and were audio recorded and transcribed for analysis. The interview centered around four key questions. The first question explored participants' perceived performance in managing their condition by exploring their specific management strategies, goal-setting practices, supportive factors, and obstacles encountered. The second question delved into their approach to challenges, decision-making processes, and the role of their support system, including healthcare providers and family. Additionally, questions were asked to explore the resources they utilized to manage their condition. Finally, participants were asked about any shifts in their perspective and management of MS after receiving the intervention.

### 2.3 Intervention

Participants received six telephone sessions of OPC (45–60 min each) across ten weeks. OPC is a structured, strength-based, solution-focused coaching intervention which comprises five integrated elements: In each OPC session, the therapist first establishes a collaborative, trusting relationship that provides a secure foundation for change. They then offer emotional support through active listening and nonjudgmental encouragement to bolster clients' motivation and persistence. Next, tailored reflective questioning and education guide clients to identify personally meaningful participation goals, recognize potential barriers, and leverage available resources. Following this, clients engage in a structured problem-solving cycle—co-designing action plans, testing them in real life, reflecting on outcomes, and refining next steps—which builds planning and monitoring skills. Finally, celebrating small, incremental successes enhances self-efficacy, reinforcing confidence and driving continued engagement with self-management.

### 2.4 Data analysis

The collected data were analyzed through an inductive thematic approach, where preliminary coding helped identify patterns and develop themes based on the emerging codes [26]. For each participant, codes and themes were identified and then compared with those from other participants to explore similar patterns or differences among them. To ensure thematic saturation—that is, the point at which additional analysis no longer produces new insights—data and coded extracts were continuously compared. This coding and analysis process was performed using NVivo 12 software [27].

The thematic analysis of the data was conducted by the first author in conjunction with the research team in four phases. In the first phase, the pre-OPC interviews were coded and analyzed, with thorough examination and comparison of codes and quotes to ensure accuracy and consistency. Themes were then developed based on the collated codes and presented to the research team for discussion. The second phase involved coding the transcripts from the final coaching sessions and post-OPC interviews, following a similar process. In the third phase, within-case comparisons were conducted where the pre-OPC data was compared with the post-OPC data for each participant. This analysis aimed to identify any changes in participants' experiences of living with MS and understand how those changes occurred. Finally, in the fourth phase, a cross-case comparison was performed to identify patterns and similarities across all participants, and note any differences that may exist. Through these four phases, the focus was on identifying themes, patterns, and changes in participants' experiences of living with MS before and after the OPC intervention.

## 3 Results

### 3.1 Participant description

This qualitative study included 10 participants who consisted of two men and eight women ranging from 24 to 60 years of age. Six participants reported to be actively involved in studying and/or paid employment, while the other Four participants were unemployed, either retired or having left their jobs due to challenges associated with their condition.

To protect participant confidentiality, each participant has been assigned a pseudonym. These pseudonyms are used consistently throughout the manuscript to discuss individual responses, results, and analysis. Further information about the characteristics of the study sample can be found in Table 1.

**Table 1:** Participants characteristics.

Participant Pseudonyms	Age	Type of MS	Years since diagnosis	Marital status	Employment status	Mobility
John	24	R/R	2	Single-Never married	Unemployed-Full time student	A
Sarah	56	S/P	6	Married or Partnership	Unemployed-Chose not to be employed	A
Michael	29	R/R	4	Married or Partnership	Employed-Full time	I
Emily	60	R/R	23	Married or Partnership	Unemployed-On disability insurance	I
David	37	R/R	21	Single-Never married	Unemployed-On disability insurance	A
Laura	48	P/P	2	Married or Partnership	Employed-Full time	A
Daniel	58	P/P	4	Widow or Widower	Retired	A
Emma	46	R/R	7	Married or Partnership	Part time-1-19 h per week	A
James	42	R/R	19	Married or Partnership	Employed-Full time	I
Olivia	41	R/R	4	Separated or divorced	Employed-Full time	I

Type of MS: R/R, relapsing remitting MS; S/P, secondary progressive MS; P/P, primary progressive MS; Employment status: Employed P/T, employed part time; Mobility: I, Independent; A, Mobile with the use of aids.

### 3.2 Themes from pre-OPC interviews

In the initial phase of the analysis, the focus was on examining the experiences of individuals living with multiple sclerosis (MS) prior to receiving the OPC intervention. The analysis of these interviews yielded four major themes, namely: resisting MS, living with MS, ongoing challenges, and strategies used in response to the challenges.

Although “Resisting MS” and “Living with MS” represent opposing stances towards the illness, they emerged as qualitatively distinct coping processes rather than simply ends of a single spectrum. “Resisting MS” captures participants' denial, avoidance, and efforts to maintain pre-MS identities despite worsening symptoms, whereas “Living with MS” reflects intentional acceptance, accommodation, and integration of the condition into daily life. Because these involve different cognitive–emotional strategies and behavioral patterns, they were retained as separate themes to highlight the nuanced ways individuals cope with MS prior to OPC.

Corresponding quotes to the themes discussed below can be found in Table 2.

**Table 2:** Themes and illustrative quotes.

Pre OPC - Theme	Pre OPC - Quote	Post OPC - Theme	Post OPC - Quote
1. Living with MS	“Then accepted my illness. Don't get me wrong, it still gets frustrating… But I mean, I might do something today and not tomorrow. And that's frustrating, but at the same time, you know, it is what it is.” (Sarah)	1. Living with MS	““I find if anything, MS is less on my mind and I feel more agency.”” (David) ““I would say I am easier on myself… as a minor setback instead so I guess the general answer is attitude is different.”“(Olivia)
2. Resisting MS	“I'm really stubborn. So, I usually go past my limits, like, almost on a daily basis…” (John) “I try not to make it a constant black mark in the mirror that I have to look at, you know, like a mole on your face … I just, I'd rather just be known for me. And I like to make sure that everything that I do around the house here is none of that is, is MS related. It's all stuff that needs to be done.” (Emily)		
3. Ongoing Challenges	““Like, if I had a question tomorrow, I would have a hard time getting in contact with them because they're so busy.”“(John) “I have a really hard time saying no to things. And so my schedule just gets over packed. And then, because I'm also a person that is committed to commitment, I won't back out of something, even if I'm not feeling well. So that is definitely, you know, something that I'm learning to deal with” (Emma) “Fatigue, brain fog, I said, I'm better than a year ago with brain fog. My ability has deteriorated extremely rapidly this year. Like, for example, this week, climbing into a boat with it, getting down to the point where things are potentially dangerous, I have to stop.” (Sarah) “I don't, I don't have a lot of people in my life that I would talk to you about a challenge like that, you know, the most important people in my life are my kids, and I don't really want to put that burden on them that I'm feeling stressed or anxious about something. So I just kind of manage it on my own. I just Just do it. Whether it works out okay or doesn't” (Daniel)	2. Ongoing Challenges	“I would say the only thing that I feel could really derail me is these kinds of lingering feelings of fatigue” (Emma) “it can be a little bit more realistic, and I certainly don't get worked up. For whatever reason, if today something isn't right and my body won't allow me to do this, it's just the way it is. Whereas prior to all this, it probably would have bothered me a whole lot more. Or certainly, you know, put me in a less than ideal mood … now, you just kind of realize that this a situation and you got to roll with the punches” (John)
4. Strategies	““Just let it be a challenge for a while and then I will process through okay, what steps can II can take… I'll break it up.”“(David) “But at the same time, you can dwell and worry about that every minute of the day. I'm certainly trying to focus on each and every day, like breaking down just to enjoy the simple little things.” (Sarah) “That if I need help, I have come to learn to ask for help or to wait for help to be offered.” (Emily)	3. Strategies	““So just keep planning. That's the key thing… Let's look ahead for a week because it's a manageable piece.”“(Laura) ““I think it's really focusing on routine… And hopefully, like if I were to fall into a time period where I kind of wasn't meeting my goals, I would feel more confident about starting up again.” “(Michael) ““…saying things out loud establishes a plan, slash motivation. So, it's probably just healthy just to talk about what you want to do out loud.”“(Sarah)

#### 3.2.1 Theme

##### 3.2.1.1 Resisting MS

Some participants expressed a strong resistance to accepting the limitations imposed by MS and were determined not to let the condition define their lives. For instance, one participant mentioned not being able to accept their limitations and constantly attempting to fulfil all their daily plans despite their limitations. While this was a common expression among several participants, other interviewees also stated that they do not want to constantly be reminded about their life with MS.

#### 3.2.2 Theme

##### 3.2.2.1 Living with MS

In contrast, a few participants reported a more accommodating approach, accepting their condition and incorporating MS into their lives. They appeared to acknowledge the reality of the illness for their lives and decided to focus on what they could control.

#### 3.2.3 Theme

##### 3.2.3.1 Ongoing challenges

Participants highlighted various ongoing challenges and struggles they faced in their lives due to MS. These challenges fell into four subthemes: lack of resources, balancing commitments/priorities, symptom-related challenges, and social challenges. Among these subthemes, resource limitations encompassed a range of challenges, such as encountering difficulties when trying to access healthcare professionals or seeking non-pharmaceutical support. Additionally, participants grappled with the complex task of balancing their commitments and priorities while accommodating their physical limitations. Symptom-related challenges primarily revolved around issues related to fatigue and mobility impairments. Another distinct set of challenges stemmed from social factors. Notably, participants encountered difficulties arising from a lack of awareness about MS among the individuals in their social circles, making it challenging to effectively convey their needs and emotions. Furthermore, the fear of becoming a burden to their families acted as a barrier to open communication and full self-expression. These diverse challenges collectively reflected the multifaceted and dynamic nature of the circumstances participants are faced with while living with MS.

#### 3.2.4 Theme

##### 3.2.4.1 Strategies

Participants developed various strategies to address the challenges they faced. These strategies encompassed resource utilization, breaking tasks into small steps, setting goals, positive focus, and asking for help.

Resource utilization primarily involved independent online research conducted by participants to discover available resources. Additionally, one participant mentioned volunteering for MS studies as a proactive means to gain information and access resources.

Participants also discussed breaking down tasks into manageable steps, setting specific goals, and maintaining a positive focus. For example, they set goals like hiking twice a week and focused on surrounding themselves with positive individuals.

The strategies that participants had developed were used to navigate some of the challenges they had mentioned. For instance, resource utilization strategies were used to compensate for the lack of access to healthcare professionals and answering their remaining questions. Furthermore, by becoming part of online MS support groups participants were able to connect with others with similar conditions and challenges and share knowledge and resources. Asking for help and setting goals around healthier diet and exercise were among the strategies used to deal with symptom-related challenges.

#### 3.2.5 Themes

##### 3.2.5.1 From Post-OPC interviews

In the second phase of the analysis, the themes and subthemes identified in the pre-OPC interviews remained mostly stable. Participants continued to report a variety of challenges. However, a few notable differences were observed. The theme of resisting MS was no longer present in the data collected from the post-OPC interviews. Additionally, new subthemes emerged under the themes of living with MS and strategies.

#### 3.2.6 Theme

##### 3.2.6.1 Living with MS

The subtheme of acceptance continued to be prominent in the post-OPC interviews. Furthermore, two new subthemes emerged: feeling more agency and a change of attitude. Participants described improved recognition of their capabilities and feeling a sense of control. Additionally, a positive change of attitude was reported by the participants. For example, a participant who resisted the use of medication to deal with symptoms in the past, adopted a new perspective and considered using medication to help with their symptom management after the OPC intervention.

#### 3.2.7 Theme

##### 3.2.7.1 Strategies

Most of the strategies discussed prior to the OPC intervention remained in use by participants in the post-OPC interviews. These included resource utilization, breaking tasks into smaller steps, setting goals, maintaining a positive focus, and asking for help. However, three additional strategies emerged in the post-OPC analysis. The first new strategy was planning ahead, which involved anticipating and accounting for the unpredictability and limitations of the condition when scheduling activities. The second strategy that emerged was being consistent, which referred to developing a routine that facilitates goal achievement. The third strategy that emerged was talking about the plan, which involved sharing and discussing plans with others for reflection and feedback.

A summary of themes and quotes for phases I and II can be found in Table 2:

### 3.3 Phase III: within case comparison of pre- and post-OPC interviews

Within case comparison of pre- and post-OPC interviews indicated that all ten participants showed some degree of change in their experience of living with MS following the OPC intervention. Each participant exhibited unique changes. For example, John became more comfortable setting boundaries and utilizing resources and Sarah became more realistic and adopted new strategies. Overall, changes were observed in balancing commitments, resource utilization, attitudes towards MS, and a sense of agency. As noted by Emily, *“I'm probably more capable than most people give me credit for... It's been an eye-opening experience.”* Daniel also indicated*, “I can achieve my goals... More open to taking medication... It just has made me a bit more confident.”*

### 3.4 Phase IV: cross-case analysis of pre- and post-OPC interviews

In the cross-case analysis of pre- and post-OPC interviews, it was observed that resistance to MS became less prominent in participants' experiences of living with the condition after the intervention. Prior to OPC, participants had developed strategies to cope with MS, and after the intervention, these strategies were reinforced and applied more effectively. Participants reported improved resource utilization and a realistic approach to goal setting. Interestingly, both participants with less resistance and those with more resistance to MS showed reinforcement of existing strategies. Additionally, participants developed new strategies, with being consistent being a common addition to their repertoire. The focus of discussions shifted from emphasizing challenges to discussing solutions and strategies, indicating a more solution-focused mindset after OPC.

There were notable changes in the discussion of challenges before and after the intervention. Post-OPC interviews revealed a decrease in the number of subthemes related to ongoing challenges in 6 out of 10 participants. While lack of resources and balancing commitments were common challenges before OPC, the emphasis shifted more towards balancing commitments and fewer participants reported lack of resources as a challenge after the intervention. Furthermore, the analysis highlighted an expansion of subthemes under the theme of living with MS post-OPC. Participants expressed a positive change in attitude, including increased realism, self-acceptance, and a sense of control over their lives.

## 4 Discussion and conclusion

### 4.1 Discussion

The objectives of this study were to increase understanding of self-management practices and challenges of individuals with MS and to explore how Occupational Performance Coaching (OPC) may influence self-management of the daily lives of people with Multiple Sclerosis (PwMS). Prior to receiving OPC, participants experienced social- and symptom-related challenges which negatively impacted engagement in daily activities as well as their self-perceptions. Additionally, participants reported that both communicating their needs and feelings with others was difficult because of the lack of knowledge and accessing professional non-pharmaceutical support were challenging. Prior studies that explored the experience of PwMS have reported similar findings of physical functioning, personal relationships, and independence being affected as the disease progresses [3,4,28]. The loss of personal independence and the ability to do what they used to do (e.g., their job, looking after a family, playing with their children) were identified as negatively impacting self-esteem and self-perception and, in some case, made them feel like a burden [28].

The theme of resisting MS reflected challenges that participants experienced in accepting the limitations caused by the disease and adjusting their lives accordingly. Difficulty coming to terms with their diagnosis and the impact it has on their lifestyle has been identified in other studies of PwMS [3,28]. Notably, adjusting outlook is identified as a strategy for managing living with MS [29]. Following receipt of OPC, resistance to the disease was not identified as a theme, suggesting that participants adjusted their outlook and changed their attitude towards themselves and their management of MS. The recognition of capabilities and increased sense of agency among the participants could have resulted from the goal-oriented and solution-based focus of OPC.

This study uncovered several strategies that participants were using to overcome daily challenges as well as those that emerged through the process of OPC. These strategies are in line with core self-management skills discussed in prior work [6,30] and align with the model of MS self-management [29]. For instance, problem-solving and decision-making have been key to developing self-management skills for people living with chronic conditions [6,30]. Breaking things down into smaller steps and setting targets were among the skills that were practiced and developed throughout the OPC intervention. As well, participants suggested enhanced ability to plan and engage in their daily activities through setting more reasonable participation goals.

Participants who had struggled with balancing priorities and recognizing their limitations when making decisions reported improvements in this regard; allowing for better decision making. They also stated that balancing commitments in daily life and asking other people for help facilitated their management of the disease. These strategies align with those for fatigue management for PwMS [31]. Furthermore, the strategy of planning ahead was used by participants to better balance their commitments and priorities. Similarly, in the MS self-management model of Ghahari et al., (2019) making arrangements in advance and preparing a plan to complete desired tasks was noted as one of the strategies under the category of setting priorities and planning.

Another strategy that was identified following OPC was being consistent with diet and physical activity to improve daily routines and better manage MS. Other studies of PwMS have noted the goal of practicing a healthy lifestyle as a self-management strategy [29].

Finding and utilizing resources is also an essential self-management skill [6]. Participants engaged in independent searching for resources and volunteered in studies as means to access resources and information. The likelihood and effectiveness by which this was done increased through the process of OPC. Overall findings from this study support the potential effectiveness of OPC for supporting development of crucial self-management skills. Future research could further explore how well participants retained their sense of agency, attitude, and strategies over time.

The findings and implications of this research should be interpreted within the limitations of this study. First, this study used a convenience sample of participants with MS who had previously agreed to be contacted for MS rehabilitation research and enrolled in the main RCT This may have resulted in recruitment of people who were motivated to make life adjustments and may have also influenced the extent of variation in the backgrounds of the participants, limiting the transferability of the findings to all PwMS. Second, due to small research team, the interventionist and interviewer in this study were the same person which may have influenced responses provided by the participants during the interviews. The interviewer used the same semi-structured interview guide both pre-and post-intervention, allowing direct within-participant comparisons, and employed prompts and follow-up questions to elicit detailed, candid reflections. Finally, it should be noted that this study was carried out during the Covid-19 pandemic. The restrictions and limitations that were imposed by the pandemic could have influenced the experiences and challenges of the participants. This may impact the extent to which the findings are transferable to other contexts. While these steps helped detect changes attributable to OPC, further studies should consider independent reviewers or triangulation methods to further strengthen reliability.

### 4.2 Innovation

This study is innovative in specifically applying Occupational Performance Coaching (OPC)—originally developed for pediatric populations [32]—to adults living with MS. This represents an expansion of OPC into a new clinical area, addressing the unique and complex self-management needs associated with MS.

Moreover, this research employs a qualitative interpretive descriptive approach that provides a more nuanced understanding of shifts in participants' experiences and perspectives before and after intervention; thereby building on the quantitative evidence to support the use of OPC with Pw MS [18]. Additionally, delivering OPC remotely via telephone increases accessibility for PwMS, and offers insight into the use of remote coaching interventions within this population.

## 5 Conclusion

In this interpretive description study of ten adults with MS, OPC appeared to shift participants away from resistance and towards a more solution-focused approach, enhancing their planning and problem-solving skills as well as their sense of agency. However, given our small, purposive sample and the dual role of the interventionist as interviewer, we do not make definitive claims about OPC's overall effectiveness or statistical generalizability. Rather, we offer rich, contextualized insights whose transferability can be judged by readers working in similar clinical settings. Future research employing larger, more diverse samples, independent data collection, and controlled designs will be essential to rigorously test OPC's efficacy and further elucidate its mechanisms of action.

## CRediT authorship contribution statement

**Niloufar Malakouti:** Writing – original draft, Software, Project administration, Methodology, Investigation, Formal analysis, Data curation, Conceptualization. **Dorothy Kessler:** Writing – review & editing, Supervision, Methodology, Formal analysis, Conceptualization. **Marcia Finlayson:** Writing – review & editing, Validation, Supervision, Conceptualization. **Samantha Stephens:** Writing – review & editing, Supervision.

## Ethical statement

This study was approved by Queen's University Health Sciences and Affiliated Teaching Hospitals Research Ethics Board (HSREB). Informed consent was obtained from all participants.

## Funding

This research was conducted as part of a clinical trial (NCT04908085) which was funded by the University Hospitals Foundation Kingston - Women's Giving Circle.

## Declaration of competing interest

The authors declare that they have no known competing financial interests or personal relationships that could have appeared to influence the work reported in this paper.
